# Software Verification with PDR: An Implementation of the State of the Art

**DOI:** 10.1007/978-3-030-45190-5_1

**Published:** 2020-03-13

**Authors:** Dirk Beyer, Matthias Dangl

**Affiliations:** 8 Johannes Kepler University, Linz, Austria; 9 University of Birmingham, Birmingham, UK; LMU Munich, Munich, Germany

**Keywords:** Software verification, Program analysis, Invariant generation, Property-directed reachability (PDR), IC3, *k*-Induction, VVT, CPAchecker

## Abstract

Property-directed reachability (PDR) is a SAT/SMT-based reachability algorithm that incrementally constructs inductive invariants. After it was successfully applied to hardware model checking, several adaptations to software model checking have been proposed. We contribute a replicable and thorough comparative evaluation of the state of the art: We (1) implemented a standalone PDR algorithm and, as improvement, a PDR-based auxiliary-invariant generator for *k*-induction, and (2) performed an experimental study on the largest publicly available benchmark set of C verification tasks, in which we explore the effectiveness and efficiency of software verification with PDR. The main contribution of our work is to establish a reproducible baseline for ongoing research in the area by providing a well-engineered reference implementation and an experimental evaluation of the existing techniques.

### 1 Introduction

Automatic software verification [24] is a broad research area with many success stories and large impact on technology that is applied in industry [2, 14, 27]. It complements other general approaches to ensure functional correctness, like software testing [31] and interactive software verification [3]. One large sub-area of automatic software verification includes algorithms and approaches that are based on SMT technology. Classic approaches like bounded model checking [10], predicate abstraction [1, 19], and $k$-induction [5, 26, 32] are well understood and evaluated; a recent survey [6] provides a uniform overview and sheds light on the differences of the algorithms. Property-directed reachability (PDR) [12] is a relatively recent (2011) approach that is not yet included in comparative evaluations that go beyond applying different implementations of the same or different techniques to a set of benchmark tasks, but additionally pair such experiments with a discussion of how the concepts can be expressed in a common formalism. The approach was originally applied to transition systems from hardware designs, but was also adapted to software verification [11, 12, 13, 15, 16, 25, 28, 29].

An extended version of this article is available as technical report [8].
A replication package is available on Zenodo [9].

While in theory, given the aforementioned body of work on the topic, the advantages and disadvantages of using PDR seem clear, we are interested in understanding the effect of applying PDR to a large set of verification tasks that were collected from academia and also from industrial software, such as the Linux kernel. To achieve this goal, we implemented one PDR adaptation for software verification, and another approach that integrates a PDR-like invariant-generation module into a $k$-induction approach.

*PDR Adaptation for Software Verification.* PDR is a model-checking algorithm that tries to construct an inductive safety invariant by incrementally learning clauses that are inductive relative to previously learned clauses. The clause-learning strategy is guided by counterexamples to induction, i.e., each time a proof of inductiveness fails, the algorithm attempts to learn a new clause to avoid the same counterexample to induction in the future. Originally, this algorithm was designed as a SAT-based technique for Boolean finite-state systems. Every adaptation of PDR to software verification therefore needs to consider how to effectively and efficiently handle the infinite state space and how to transfer the algorithm from SAT to SMT. Furthermore, the adaptation to software has to deal with the program counter.

*PDR-like Invariant Generation.* Whenever an induction-proof attempt fails with a counterexample, the counterexample describes a state $s$ that can transition into a bad state (that violates the safety property), which means that in order to make the proof succeed, $s$ must be removed from consideration by an auxiliary invariant. From this bad-state predecessor $s$, the clause-learning strategy of PDR proceeds to generate such an auxiliary invariant by applying the following two steps: (1) $s$ is first generalized to a set of states $C$ that all transition into a bad state; (2) an invariant is constructed that is (a) inductive relative to previously found invariants[1] and (b) at least strong enough to eliminate all states in $C$. If it fails to construct such an invariant and prove its inductiveness, then the steps are recursively re-applied to the counterexample obtained from the failed induction attempt.

We experimentally investigate two implementations of adaptations of PDR to software verification (CPAchecker-CTIGAR and Vvt-CTIGAR), as well as several combinations that use the PDR-like invariant-generation module that we designed and implemented for this study.

**Example.** Figure 1 shows an example C program (`eq2.c`) that contains four unsigned integer variables `w`, `x`, `y`, and `z`. In line `10`, the variable `w` is initialized to an unknown value via the input function `__VERIFIER_nondet_uint()`; then, its value is copied to `x` in line `11`. In line `12`, variable `y` is initialized with the value of `w + 1`, and in line `13`, variable `z` is initialized with the value of `x + 1`, such

[1] An assertion $F$ is said to be inductive relative to an invariant *Inv* if *Inv* can be used as an auxiliary invariant for the proof of inductiveness $\forall s_j, s_{j+1} : F(s_j) \land T(s_j, s_{j+1}) \Rightarrow F(s_{j+1})$ by conjoining *Inv* to the induction hypothesis $F(s_j)$, such that the modified induction query $\forall s_j, s_{j+1} : F(s_j) \boldsymbol{\land} \boldsymbol{Inv}(\mathbf{s_j}) \land T(s_j, s_{j+1}) \Rightarrow F(s_{j+1})$ allows a proof by induction to succeed. [12]

```
1  extern void __VERIFIER_error() __attribute__
 ↪ ((__noreturn__));
2  extern unsigned int __VERIFIER_nondet_uint(void);
3  void __VERIFIER_assert(int cond) {
4    if (!(cond)) {
5      ERROR: __VERIFIER_error();
6    }
7    return;
8  }
9  int main(void) {
10   unsigned int w = __VERIFIER_nondet_uint();
11   unsigned int x = w;
12   unsigned int y = w + 1;
13   unsigned int z = x + 1;
14   while (__VERIFIER_nondet_uint()) {
15     y++;
16     z++;
17   }
18   __VERIFIER_assert(y == z);
19   return 0;
20 }
```

Fig. 1: Example C program `eq2.c`

that at this point, `w` and `x` are equal to each other, and `y` and `z` are also equal to each other. Then, from line `14` to line `17`, a loop with a nondeterministic exit condition (and therefore an unknown number of iterations) increments in each iteration both variables `y` and `z`. Lastly, line `18` asserts that after the loop, `y` and `z` are (still) equal to each other. Since `y` and `z` are equal before the loop, and are always incremented together within the loop, the invariant $y = z$ is inductive. However, since there is no direct connection between `y` and `z` but only an indirect one via their shared dependency on `w`, naïve data-flow-based techniques may fail to find this invariant. In fact, we tried several configurations of the verification framework CPAchecker, and found that many of them fail to prove this program:

- Plain $k$-induction without auxiliary-invariant generation fails, because it never checks if $y = z$ is a loop invariant and instead only checks the reachability of the assertion failure (located after loop). The reachability of the assertion failure, in turn, depends on the nondeterministic loop-exit condition. Therefore we cannot conclude from "the assertion failure was not reached in $k$ previous iterations" that "the assertion failure cannot be reached in the next iteration": In the absence of auxiliary invariants, a valid counterexample to this induction hypothesis would always be that in the previous iterations the assertion *condition* was in fact violated and an assertion *failure* was not reached only *because the loop was not exited.*
- A data-flow analysis based on the abstract domain of Boxes [21] fails, because it is not able to track variable equalities.
- A data-flow analysis based on a template Eq for tracking the equality of pairs of variables fails, because while it detects the invariant $w = x$, it is unable to make the step to $y = z$ due to the inequalities between `w` and `y`, and `x` and `z`, respectively.
- For consistency with our evaluation, we also applied a data-flow analysis based on a template for tracking whether a variable is even or odd; obviously this is not useful for this program, and thus, this configuration also fails.
- Even combining the previous three techniques into a compound invariant generator that computes auxiliary invariants for $k$-induction does not yield a successful configuration for this verification task.
- The invariant generator KIPDR (the above-mentioned adaptation of PDR to $k$-induction, which we present in more detail in Sect. 3), however, detects the invariant $y = z$ and is therefore able to construct a proof by induction for this verification task.

We will now briefly sketch how KIPDR detects the invariant $y = z$ for the example verification task. At first, KIPDR attempts to prove by induction that when line `18` is reached, the assertion condition holds, which fails as discussed previously. However, this failed induction attempt yields a counterexample to induction where the values of `y` and `z` differ from each other, e.g., $y = 0 \wedge z = 1$, which is then generalized to $y \neq z$, i.e., a set of states that includes the concrete predecessor of a bad state from the counterexample, as well as many other states that would violate the assertion, if they were reachable themselves. Then, KIPDR attempts to find an inductive invariant that eliminates all of these states, and the attempt succeeds with the invariant $y = z$. Afterwards, KIPDR re-attempts its original induction proof to show that the assertion is never violated, which now succeeds due to the auxiliary invariant $y = z$.

**Contributions.** We present the following contributions:

- We implement one adaptation of PDR to software verification (based on [11, 20]) in the open-source verification framework CPAchecker, in order to establish a baseline for comparison with new ideas for improvement.
- We design and implement the algorithm KIPDR, as a new module for invariant generation that is based on ideas from PDR and use this module as an extension to a state-of-the-art approach to $k$-induction [5].
- We conduct a large experimental study to compare several tools and approaches to software verification using PDR as a component, to highlight strengths and weaknesses of PDR in the domain of software verification.
- We contribute a set of small examples that need invariants that are more difficult to obtain for standard data-flow-based approaches than the invariants necessary for programs in the large benchmark set.

**Related Work.** While PDR (also known as IC3 for its first implementation [12]) was introduced as a SAT-based algorithm for model checking finite-state Boolean transition systems [13], several approaches have since then been presented to extend it to SMT and to apply it to the verification of software models: PDR has been suggested as an interpolation engine for Impact, but experiments have shown that it is too expensive in the general case, and is most effective if only applied as a fall-back engine for cases where a cheaper interpolation engine fails to produce useful interpolants [15]. It also has been proposed to improve this approach by tracking control-flow locations explicitly instead of symbolically [28], thereby avoiding the problem that many iterations of the algorithm are spent only to learn the control flow, and this idea has later been extended by several improvements to the generalization step of PDR [29]. Another approach is to model the program using a Boolean abstraction, which has the advantage that it requires only few changes to the original algorithm, but the disadvantage that a refinement procedure is necessary to handle the spurious paths introduced by the abstraction: One such approach uses infeasible error paths (i.e., counterexample-guided abstraction refinement (CEGAR) [17]) to refine the abstraction [16], while another (CTIGAR) uses counterexamples to induction [11]; both of these refinement techniques use interpolation to obtain abstraction predicates; the latter of the two techniques is used in two of the configurations we compare in our evaluation (CPAchecker-CTIGAR and Vvt-CTIGAR [20]). A different extension of PDR to verify infinite-state systems that does not require abstraction refinement is property-directed $k$-induction [25], which increases the power of the induction checks used in PDR by applying $k$-induction instead of 1-induction, and which uses model-based generalization in addition to interpolation to reason about potentially-infinite sets of states. Unfortunately, support for effective model-based generalization is rare in SMT solvers [2], making this approach impractical. In contrast, our KIPDR algorithm presented in Sect. 3 only requires support for interpolation, which is available in several SMT solvers.

Despite this multitude of adaptations of PDR to infinite-state systems, most implementations in practice require their input to be encoded as transition systems. The only available software verifiers applicable to actual C programs and implement PDR-based techniques are CPAchecker [7], SeaHorn [23], and Vvt [20].

### 2 Background

In this section, we briefly introduce the algorithms PDR and $k$-induction, which provide the core concepts on which we base our ideas. In the following description of PDR and $k$-induction, we use the following notation: given the state variables $s$ and $s'$ within a state-transition system $T$ that represents the program, predicate $I(s)$ denotes that $s$ is an initial state, $T(s, s')$ that a transition from $s$ to $s'$ exists, and $P(s)$ that the safety property $P$ holds for state $s$.

#### 2.1 PDR

PDR maintains a list of $k$ frames, where a frame $F_i$ is a predicate that represents an overapproximation of all states reachable within at most $0 \leq i \leq k$ steps, and a queue of proof obligations, which guide invariant discovery towards invariants

[2] The implementation of the approach of property-directed $k$-induction combines two SMT solvers, because neither of them supports all features required by the technique.

relevant to prove the correctness of a safety property $P$. For a given state $s$, the notation $F_i(s)$ means that the predicate $F_i$ holds for state $s$. The index $i$ of a frame $F_i$ is called its *level*, and the frame $F_k$ is called the *frontier*, because it represents the largest overapproximation of reachable states computed by the algorithm [12]. The algorithm maintains the following invariants:

1. $F_0(s) = I(s)$, i.e., the first frame represents precisely the initial states.
2. $\forall i \in \{0, \ldots, k\} : F_i(s) \Rightarrow P(s)$, i.e., every frame contains only states that satisfy the safety property.
3. $\forall i \in \{0, \ldots, k-1\} : F_i(s) \Rightarrow F_{i+1}(s)$, i.e., a frame $F_{i+1}$ represents in addition such states that are reachable with $i+1$ steps.
4. $\forall i \in \{0, \ldots, k-1\} : F_i(s) \wedge T(s, s') \Rightarrow F_{i+1}(s')$, i.e., each frame is inductive relative to its predecessor.

Using these data structures and algorithm invariants, the algorithm attempts to find either a counterexample to $P$ or a 1-inductive invariant $F_i$ such that $F_i(s) \Leftrightarrow F_{i+1}(s)$ for some level $i \in \{0, \ldots, k-1\}$. Until either of these potential outcomes is reached, PDR shifts back and forth between the following two phases:

1. If the set of states represented by the frontier $F_k$ does not contain any predecessor states of $\neg P$-states (i.e., $\forall s_j, s_{j+1} : F_k(s_j) \wedge T(s_j, s_{j+1}) \Rightarrow P(s_{j+1})$, called frontier-incrementation check), a new frontier $F_{k+1}$ is created and initialized to $P$. Subsequently, the algorithm attempts to push forward [3] each predicate $c$ of each frame $F_i$ with $0 \leq i \leq k$ for which the consecution check $F_i(s_j) \wedge T(s_j, s_{j+1}) \Rightarrow c(s_{j+1})$ holds (see Fig. 2a). If, on the other hand, the frontier-incrementation check fails, PDR extracts a $\neg P$-predecessor $t$ in $F_k$, which represents a counterexample to induction (CTI), from the failed query as proof obligation $\langle t, k-1 \rangle$ (see Fig. 2b, top).
2. While the queue of proof obligations is not empty, PDR processes the queue by trying to prove for each proof obligation $\langle t, i \rangle$ that the CTI-state $t$ is itself not reachable from $F_i$ and therefore does not need to be considered as a relevant $\neg P$-predecessor. For this proof, PDR chooses some predicate $c \Rightarrow \neg t$ with $\forall s : F_i(s) \Rightarrow c(s)$. PDR then checks if $c$ is inductive relative to $F_i$ by performing the consecution check $F_i(s_j) \wedge c(s_j) \wedge T(s_j, s_{j+1}) \Rightarrow c(s_{j+1})$. If the consecution check succeeds, the frames $F_1, \ldots, F_{i+1}$ can be strengthened by adding $c$, thus ruling out the CTI $t$ in these frames for the future (see Fig. 2b, left). Also, unless $i = k$, we add a new proof obligation $\langle t, i+1 \rangle$ to the queue as an optimization to initiate forward propagation, because we expect that the CTI-state $s$ would otherwise be rediscovered later at a higher level [11]. Otherwise, i.e., the consecution check does *not* succeed for clause $c$, the algorithm extracts a predecessor $u$ of $t$ from the failed consecution check, which is added as a new proof obligation $\langle u, i-1 \rangle$ if $i > 0$ and $t \wedge I$ is unsatisfiable (see Fig. 2b, right). Otherwise, $u$ represents the initial state of a real counterexample to $P$.

An example of this algorithm is presented in a technical report [8, pp. 7–8]. A more detailed presentation of PDR can be found in the literature [12].

[3] By "push forward", we mean to add a predicate $c$ from frame $F_i$ to frame $F_{i+1}$ [12].

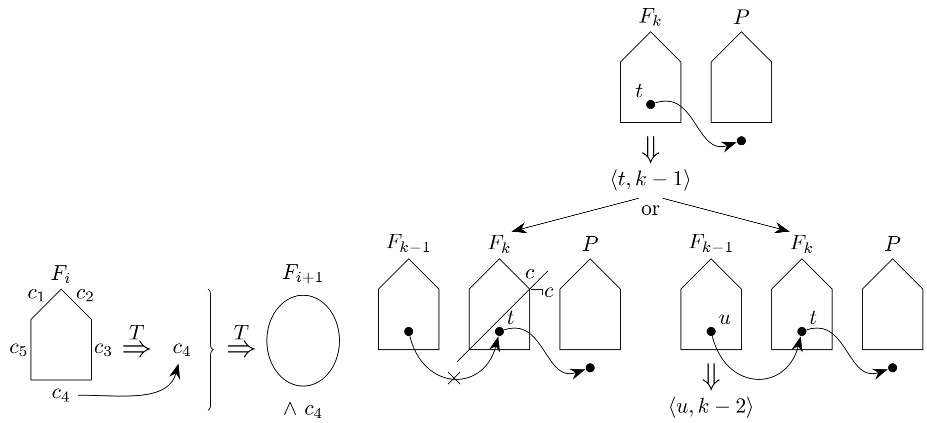

**(a)** Consecution check makes sure to only conjoin to frame $F_{i+1}$ such $c_i$ from $F_i$ that are inductive relative to $F_i$ w.r.t. transition relation $T$

**(b)** If phase 1 results in a proof obligation $\langle t, k-1 \rangle$ (top), then phase 2 resolves either by strengthening $F_k$ with $c$ (left), or by creating a new (backwards) proof obligation $\langle u, k-2 \rangle$ (right); if the chain of proof obligations propagates back to the initial states, then a feasible error path is found

Fig. 2: Visualization of (a) the consecution check and (b) the handling of proof-obligations.

### 2.2 *k*-Induction

Like PDR, $k$-induction attempts to prove a safety property $P$ by applying induction. However, while PDR strengthens its induction hypothesis by using clauses extracted from specific counterexamples to induction after failed induction attempts, $k$-induction strengthens its induction hypothesis by increasing the length of the unrolling of the transition relation.

Starting with an initial value for the bound $k$ (usually 1), the $k$-induction algorithm increases the value of $k$ iteratively after each unsuccessful attempt at finding a specification violation (base case), proving correctness via complete loop unrolling (forward condition), or inductively proving correctness of the program (inductive-step case).

**Base Case.** The base case of $k$-induction consists of running BMC with the current bound $k$.[4] This means that starting from all initial program states, all

[4] We define the loop bound as the number of visits of the loop head, that is, with loop bound $k = 1$, the loop head is visited once, but there was not yet any unwinding of the loop body. This nicely matches the intuition for $k$-induction: 1-inductiveness means that if the invariant holds for one state (without loop unrolling), then it holds again after one loop unrolling in the successor state; $k$-inductiveness means that if the invariant holds for $k$ states ($k-1$ loop unrollings), then it holds again after one more loop unrolling in the successor state.

states of the program reachable within at most $k-1$ unwindings of the transition relation are explored. If a $\neg P$-state is found, the algorithm terminates.

**Forward Condition.** If no $\neg P$-state is found by the BMC in the base case, the algorithm continues by performing the forward-condition check, which attempts to prove that BMC fully explored the state space of the program by checking that no state with distance $k' > k-1$ to the initial state is reachable. If this check is successful, the algorithm terminates.

**Inductive-Step Case.** The forward-condition check, however, can only prove safety for programs with finite (and, in practice, short) loops. To prove safety beyond the bound $k$, the algorithm applies induction: The inductive-step case attempts to prove that after every sequence of $k$ unrollings of the transition relation that did not reach a $\neg P$-state, there can also be no subsequent transition into a $\neg P$-state by unwinding the transition relation once more. In the realm of model checking of software, however, the safety property $P$ is often not directly $k$-inductive for any value of $k$, thus causing the inductive-step-case check to fail. It is therefore state-of-the-art practice to add auxiliary invariants to this check to further strengthen the induction hypothesis and make it more likely to succeed. Thus, the inductive-step case proves a program safe if the following condition is unsatisfiable:

$$Inv(s_n) \wedge \bigwedge_{i=n}^{n+k-1} (P(s_i) \wedge T(s_i, s_{i+1})) \wedge \neg P(s_{n+k})$$

where $Inv$ is an auxiliary invariant, and $s_n, \ldots, s_{n+k}$ is any sequence of states. If this check fails, the induction attempt is inconclusive, and the program is neither proved safe nor unsafe yet with the current value of $k$ and the given auxiliary invariant. In this case, the algorithm increases the value of $k$ and starts over.

A detailed presentation of $k$-induction can be found in the literature [5, 6].

### 3 Combining $k$-Induction with PDR

Algorithm 1 shows an extension of $k$-induction with continuously-refined invariants [5] that applies PDR's aspect of learning from counterexamples to induction and that can be applied both as a main proof engine as well as an invariant generator. This allows us to apply this extension of $k$-induction as an invariant generator to a main $k$-induction procedure, similar to the KI←⟲–KI approach [5].

**Inputs.** The algorithm takes the following inputs: The value $k_{init}$ is used to initialize the unrolling bound $k$, whereas the function inc is used to increase $k$ in line 33 after each major iteration of the algorithm, up to an upper limit of $k$ defined by the value $k_{max}$ enforced in line 3. The set of initial program states is described by the predicate $I$, the possible state transitions are described

**Algorithm 1** Iterative-Deepening $k$-Induction with Property Direction

**Input:** the initial value $k_{init} \geq 1$ for the bound $k$,
an upper limit $k_{max}$ for the bound $k$,
a function $\mathsf{inc} : \mathbb{N} \to \mathbb{N}$ with $\forall n \in \mathbb{N} : \mathsf{inc}(n) > n$,
the initial states defined by the predicate $I$,
the transfer relation defined by the predicate $T$,
a safety property $P$,
a function get_currently_known_invariant to obtain auxiliary invariants,
a Boolean *pd* that enables or disables property direction,
a function $\mathsf{lift} : \mathbb{N} \times (S \to \mathbb{B}) \times (S \to \mathbb{B}) \times S \to (S \to \mathbb{B})$, and
a function $\mathsf{strengthen} : \mathbb{N} \times (S \to \mathbb{B}) \times (S \to \mathbb{B}) \to (S \to \mathbb{B})$,
where $S$ is the set of program states.
**Output:** **true** if $P$ holds, **false** otherwise
**Variables:** the current bound $k := k_{init}$,
the invariant $\mathit{InternalInv} := \mathit{true}$ computed by this algorithm internally, and
the set $O := \{\}$ of current proof obligations.

**while** $k \leq k_{max}$ **do**
  $O_{prev} := O$
  $O := \{\}$
  $\mathit{base\_case} := I(s_0) \wedge \bigvee_{n=0}^{k-1} \left( \bigwedge_{i=0}^{n-1} T(s_i, s_{i+1}) \wedge \neg P(s_n) \right)$
  **if** $\mathsf{sat}(\mathit{base\_case})$ **then**
    **return false**
  $\mathit{forward\_condition} := I(s_0) \wedge \bigwedge_{i=0}^{k-1} T(s_i, s_{i+1})$
  **if** $\neg\,\mathsf{sat}(\mathit{forward\_condition})$ **then**
    **return true**
  **if** *pd* **then**
    **for each** $o \in O_{prev}$ **do**
      $\mathit{base\_case}_o := I(s_0) \wedge \bigvee_{n=0}^{k-1} \left( \bigwedge_{i=0}^{n-1} T(s_i, s_{i+1}) \wedge \neg o(s_n) \right)$
      **if** $\mathsf{sat}(\mathit{base\_case}_o)$ **then**
        **return false**
      **else**
        $\mathit{step\_case}_{o_n} := \bigwedge_{i=n}^{n+k-1} (o(s_i) \wedge T(s_i, s_{i+1})) \wedge \neg o(s_{n+k})$
        $\mathit{ExternalInv} := \mathsf{get\_currently\_known\_invariant}()$
        $\mathit{Inv} := \mathit{InternalInv} \wedge \mathit{ExternalInv}$
        **if** $\mathsf{sat}(\mathit{Inv}(s_n) \wedge \mathit{step\_case}_{o_n})$ **then**
          $s_o :=$ satisfying predecessor state
          $O := O \cup \{\neg \mathsf{lift}(k, \mathit{Inv}, o, s_o)\}$
        **else**
          $\mathit{InternalInv} := \mathit{InternalInv} \wedge \mathsf{strengthen}(k, \mathit{Inv}, o)$
  $\mathit{step\_case}_n := \bigwedge_{i=n}^{n+k-1} (P(s_i) \wedge T(s_i, s_{i+1})) \wedge \neg P(s_{n+k})$
  $\mathit{ExternalInv} := \mathsf{get\_currently\_known\_invariant}()$
  $\mathit{Inv} := \mathit{InternalInv} \wedge \mathit{ExternalInv}$
  **if** $\mathsf{sat}(\mathit{Inv}(s_n) \wedge \mathit{step\_case}_n)$ **then**
    **if** *pd* **then**
      $s :=$ satisfying predecessor state
      $O := O \cup \{\neg \mathsf{lift}(k, \mathit{Inv}, P, s)\}$
  **else**
    **return true**
  $k := \mathsf{inc}(k)$
**return unknown**

by the transition relation $T$, and the set of safe states is described by the safety property $P$. The accessor get_currently_known_invariant is used to obtain the strongest invariant currently available via a concurrently running (external) auxiliary-invariant generator. A Boolean flag *pd* (reminding of "property-directed") is used to control whether or not failed induction checks are used to guide the algorithm towards a sufficient strengthening of the safety property $P$ to prove correctness; if *pd* is set to *false*, the algorithm behaves exactly like standard $k$-induction. Given a failed attempt to prove some candidate invariant $Q$[5] by induction, the function lift is used to obtain from a concrete counterexample-to-induction (CTI) state a set of CTI states described by a state predicate $C$. An implementation of the function lift needs to satisfy the condition that for a CTI $s \in S$ where $S$ is the set of program states, $k \in \mathbb{N}$, $Inv \in (S \to \mathbb{B})$, $Q \in (S \to \mathbb{B})$, and $C = \mathsf{lift}(k, Inv, Q, s)$, the following holds:

$$C(s) \wedge \left( \forall s_n \in S : C(s_n) \;\Rightarrow\; Inv(s_n) \wedge \bigwedge_{i=n}^{n+k-1} (Q(s_i) \wedge T(s_i, s_{i+1})) \Rightarrow \neg Q(s_{n+k}) \right),$$

which means that the CTI $s$ must be an element of the set of states described by the resulting predicate $C$ and that all states in this set must be CTIs, i.e., they need to be $k$-predecessors of $\neg Q$-states, or in other words, each state in the set of states described by the predicate $C$ must reach some $\neg Q$-state via $k$ unrollings of the transition relation $T$. We can implement lift using Craig interpolation [18, 30] between $A : s = s_n$ and $B : Inv(s_n) \wedge \bigwedge_{i=n}^{n+k-1} (Q(s_i) \wedge T(s_i, s_{i+1})) \Rightarrow \neg Q(s_{n+k})$, because $s$ is a CTI, and therefore we know that $A \Rightarrow B$ holds. [6] Hence, the resulting interpolant satisfies the criteria for $C$ to be a valid lifting of $s$ according to the requirements towards the function lift as outlined above. The function strengthen is used to obtain for a $k$-inductive invariant a stronger $k$-inductive invariant, i.e., its result needs to imply the input invariant, and, just like the input invariant, it must not be violated within $k$ loop iterations and must be $k$-inductive.

**Algorithm.** Lines 4 to 6 show the base-case check (BMC) and lines 7 to 9 show the forward-condition check, both as described in Sect. 2. If *pd* is set to *true*, lines 10 to 23 attempt to prove each proof obligation using $k$-induction: Lines 12 to 14 check the base case for a proof obligation $o$. If any violations of the proof obligation $o$ are found, this means that a predecessor state of a $\neg P$-state, and thus, transitively, a $\neg P$-state, is reachable, so we return *false*. If, otherwise, no violation was found, lines 16 to 23 check the inductive-step case to prove $o$.[7] We strengthen the induction hypothesis of the step-case check by

[5] Depending on the step the algorithm is in, $Q$ may be either the safety property $P$ or a proof obligation $o$.

[6] The formula $C$ is called Craig interpolant for two formulas $A$ and $B$ with $A \Rightarrow B$, if $A \Rightarrow C$, $C \Rightarrow B$, and all variables in $C$ occur in both $A$ and $B$.

[7] Note that we do not need to check the forward condition for proof obligations, because the forward condition is unrelated to the safety property and the proof obligations, and therefore only needs to be checked once in each major iteration (i.e., once after each increment of $k$).

conjoining auxiliary invariants from an external invariant generator (via a call to get_currently_known_invariant) and the auxiliary invariant computed internally from proof obligations that we successfully proved previously. If the step-case check for $o$ is unsuccessful, we extract the resulting CTI state, lift it to a set of CTI states, and construct a new proof obligation so that we can later attempt to prove that these CTI states are unreachable. If, on the other hand, the step-case check for $o$ is successful, we no longer track $o$ in the set $O$ of unproven proof obligations (this case corresponds to line 22). We could now directly use the proof obligation as an invariant, but instead, in line 23 we first try to strengthen it into a stronger invariant that removes even more unreachable states from future consideration before conjoining it to our internally computed auxiliary invariant. In our implementation, we implement strengthen by attempting to drop components from a (disjunctive) invariant and checking if the remaining clause is still inductive. In lines 24 to 32, we check the inductive-step case for the safety property $P$. This check is mostly analogous to the inductive-step case check for the proof obligations described above, except that if the check is successful, we immediately return *true*.

Note that Alg. 1 eagerly increases $k$, even if the set $O$ of proof obligations is not empty. This heuristic prevents the PDR part from iterating through long chains of proof obligations, it rather delegates the unrolling to the $k$-induction part.

An in-depth discussion of a practical example of Alg. 1 is presented in a technical report [8, pp. 12–14].

## 4 Evaluation

In this section, we present an extensive experimental study on the effectiveness and efficiency of adaptations of PDR to software verification.

### 4.1 Compared Approaches

We use the following abbreviations to distinguish between the different techniques that we evaluated:

**CTIGAR:** CTIGAR [11] is an adaptation of PDR to software verification. Our evaluation compares two implementations of CTIGAR, namely Vvt-CTIGAR from the tool Vvt and our own implementation CPAchecker-CTIGAR. Vvt [20] also provides a configuration that runs a parallel portfolio combination of Vvt-CTIGAR and bounded model checking, which we call Vvt-Portfolio.

**KI:** KI [5] denotes the plain $k$-induction algorithm without property direction and without auxiliary invariants, i.e., we configure Alg. 1 such that $pd = false$ and get_currently_known_invariant() always returns *true*.

**KIPDR:** KIPDR denotes a configuration of Alg. 1 such that $pd = true$ and get_currently_known_invariant() always returns *true*, i.e., $k$-induction with property direction but without additional auxiliary-invariant generation. KIPDR is, like CTIGAR, an adaptation of PDR to software verification.

**KI←↺DF:** KI←↺DF [5] denotes a parallel combination of $k$-induction (without property direction) with a data-flow-based auxiliary-invariant generator that continuously supplies the $k$-induction procedure with invariants. Here, we configure Alg. 1 such that $pd = false$ and get_currently_known_invariant() always returns the most recent (strongest) invariant computed by the data-flow-based auxiliary-invariant generator.

**KI←↺KIPDR:** Similarly to KI←↺DF, KI←↺KIPDR denotes a parallel combination of $k$-induction with an auxiliary-invariant generator — in this case, KIPDR — that continuously supplies invariants to the $k$-induction procedure. Here, we configure one instance of Alg. 1 such that $pd = false$ and get_currently_known_invariant() always returns the most recent (strongest) invariant computed by KIPDR (a second instance of Alg. 1 that is configured such that $pd = true$ and get_currently_known_invariant() always returns *true*).

**KI←↺DF;KIPDR** KI←↺DF;KIPDR denotes a parallel combination of $k$-induction with an auxiliary-invariant generator that uses a sequential combination of a data-flow-based invariant generator and KIPDR to continuously supply $k$-induction with auxiliary invariants. We configure one instance of Alg. 1 such that $pd = false$ and get_currently_known_invariant() always returns the most recent (strongest) invariant computed by a sequential combination of the data-flow-based invariant generator and KIPDR (a second instance of Alg. 1 that runs after the invariant generator finishes and is configured such that $pd = true$ and get_currently_known_invariant() always returns *true*).

We do not evaluate the used invariant generators as standalone approaches, as they are designed specifically to be used as auxiliary components and do not perform well enough in isolation. For example, data-flow based invariant-generation approaches are often too imprecise to verify tasks, whereas more precise techniques like KIPDR might run into too many timeouts to be competitive. Instead, we use the framework of $k$-induction with continuously refined invariant generation, which has been shown to be able to combine quick and precise techniques [5].

#### 4.2 Experimental Setup

Details about the experimental setup can be found in the technical report [8], which describes in Sect. 4.2 which tool versions and SMT theory we used, in Sect. 4.3 which benchmark sets we used and why, in Sect. 4.4 which existing verifiers we compared to and which versions we took, in Sect. 4.5 which computing resources and execution environment were used, in Sect. 4.6 the scoring schema, and in Sect. 4.12 which threats to the validity of the evaluation we identified and how we mitigated them.

#### 4.3 Results

In the following, we pick a few highlights from the results of our experimental evaluation, in order to illustrate the potential of the approaches. A complete and more detailed report of the results is available in the extended version of this article [8].

Table 1: Results for all 5 591 verification tasks, 1 457 of which contain bugs, while the other 4 134 are considered to be safe, for the two CTIGAR implementations CPAchecker-CTIGAR and Vvt-CTIGAR, for a theoretical "virtual best" combination of both CTIGAR implementations where an oracle selects the best implementation for each task, for *k*-induction without auxiliary invariants (KI), and for the best configurations of each tool: CPAchecker's KI←⟲-DF;KIPDR, SeaHorn, and Vvt as a portfolio verifier.

| Verifier | CTIGAR | | KI | Best of each tool | | |
|---|---|---|---|---|---|---|
| | CPAchecker | Vvt | | KI←⟲-DF; KIPDR | SeaHorn | Vvt - Portfolio |
| Score | 1 903 | 879 | 3 282 | **5 398** | 2 848 | 727 |
| Correct results | 1 087 | 739 | 2 075 | 3 095 | 3 468 | 839 |
| Correct proofs | 832 | 524 | 1 239 | 2 335 | 2 724 | 528 |
| Correct alarms | 255 | 215 | 836 | 760 | 744 | 311 |
| Wrong proofs | **0** | 5 | **0** | **0** | 46 | 9 |
| Wrong alarms | 1 | 14 | 2 | 2 | 117 | 22 |
| Timeouts | 3 982 | 110 | 2 764 | 2 006 | 1 476 | 524 |
| Out of memory | 23 | 28 | 315 | 243 | 231 | 22 |
| Other inconclusive | 498 | 4 695 | 435 | 245 | 253 | 4 175 |
| Times for correct results | | | | | | |
| Total CPU Time (h) | 9.0 | 3.2 | 30 | 54 | 29 | 5.7 |
| Mean CPU Time (s) | 30 | 16 | 52 | 63 | 31 | 25 |
| Median CPU Time (s) | 4.9 | 0.24 | 9.8 | 10 | 0.89 | 0.45 |

**Suitability of CPAchecker for PDR.** The first set of experiments showed that our implementation is at least as good as (and even better than) the only available implementation of PDR for software model checking. Columns two and three of Table 1 compare the results obtained by running the two implementations of CTIGAR on the whole benchmark set, and the last column of the table shows the results achieved with the standard configuration of Vvt, which runs not only CTIGAR, but a portfolio analysis of CTIGAR and bounded model checking. The quantile plot in Fig. 3 shows the CPU times that the two tool configurations spent on their correct results.

**KIPDR versus Data-Flow Techniques.** Data-flow-based techniques are usually more efficient than KIPDR. The higher efficiency of data-flow-based techniques is most likely due to the simple form of the invariants needed to prove the programs correct. In order to experiment with progams that have some more interesting invariants, we created a few programs by hand and tried to verify those. Table 2 shows the results we obtained for these tasks. Our experiments support the hypothesis that KIPDR can be very strong and efficient on tasks that other approaches can not solve. It is important to note that this is an 'exists' statement and can not be generalized, as shown by the results that KIPDR is often outperformed by simpler, data-flow-based invariant-generation techniques.

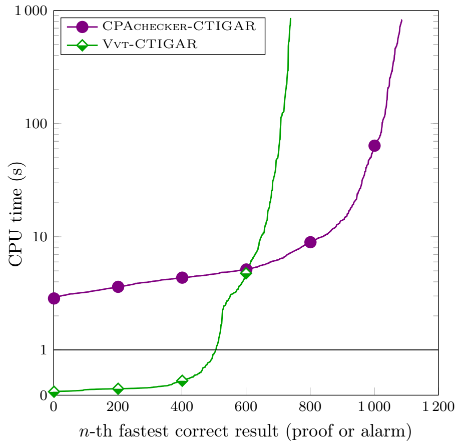

Fig. 3: Comparing two implementations of CTIGAR; quantile plot for accumulated number of solved tasks (proofs and alarms) showing the CPU time (linear scale below 1 s, logarithmic above) for the successful results of CPAchecker-CTIGAR and Vvt-CTIGAR

Table 2: Results of four $k$-induction-based configurations in CPAchecker with different approaches for generating auxiliary invariants for seven manually crafted verification tasks that do not contain bugs and are not solved by $k$-induction without auxiliary invariants; an entry "T" means that the CPU-time limit was exceeded, an entry "M" means that the memory limit was exceeded, and all other entries represent the CPU time a configuration spent to correctly solve the task

| Task | KI←DF | | | KI↚-KIPDR |
|---|---|---|---|---|
| | Boxes | Boxes, Eq | Boxes, Eq, Mod2 | |
| const.c | 3.3 s | 3.3 s | **3.2 s** | 3.8 s |
| eq1.c | T | **3.2 s** | 3.3 s | 4.9 s |
| eq2.c | M | M | M | **3.9 s** |
| even.c | T | T | **3.5 s** | 3.9 s |
| odd.c | T | T | **3.4 s** | 4.1 s |
| mod4.c | T | T | T | **3.6 s** |
| bin-suffix-5.c | M | M | M | **3.6 s** |

**Comparison with Non-PDR Approaches.** The seven example programs [8] were added to the benchmark collection that was also used for SV-COMP 2019, and thus, results are available for all verifiers that participated in the competition [9]. Table 3 summarizes the results of the best six verifiers in comparison with the KI↚-KIPDR approach that we created for the study in this paper. Those verifiers are, in alphabetical order, Skink, Ultimate Automizer, Ultimate Kojak,

[8] https://github.com/sosy-lab/sv-benchmarks/tree/svcomp19/c/loop-invariants/

[9] See the last seven rows in this table: https://sv-comp.sosy-lab.org/2019/results/results-verified/ReachSafety-Loops.table.html

Table 3: Results of SV-COMP 2019 for the six verifiers that performed best on our seven manually crafted verification tasks, compared to the results of KI⇜-KIPDR approach previously shown in Table 2; an entry "T" means that the CPU-time limit was exceeded, an entry "M" means that the memory limit was exceeded, an entry "O" means that the verifier gave up deliberately for other reasons, and all other entries represent the CPU time a verifier configuration spent to correctly solve the task; note that SV-COMP 2019 used Ubuntu 18.04 based on Linux 4.15, whereas our evaluation of KI⇜-KIPDR used Ubuntu 16.04 based on Linux 4.4; otherwise, the evaluation environment was the same

| Task | SV-COMP 2019 | | | | | | KI⇜-KIPDR |
|---|---|---|---|---|---|---|---|
| | Skink | UAutomizer | UKojak | UTaipan | VeriAbs | VIAP | |
| `const.c` | 4.2 s | 8.7 s | 9.1 s | 8.2 s | 13 s | 110 s | **3.8 s** |
| `eq1.c` | 290 s | 7.8 s | 7.6 s | 8.3 s | 14 s | 57 s | **4.9 s** |
| `eq2.c` | 4.1 s | 8.1 s | 8.6 s | 7.6 s | 14 s | 4.7 s | **3.9 s** |
| `even.c` | **3.7 s** | 7.4 s | 8.2 s | 8.6 s | 140 s | 4.5 s | 3.9 s |
| `odd.c` | O | 9.6 s | T | 11 s | 140 s | 4.6 s | **4.1 s** |
| `mod4.c` | 4.0 s | 8.4 s | 8.4 s | 7.7 s | 140 s | 4.5 s | **3.6 s** |
| `bin-suffix-5.c` | O | 14 s | T | 13 s | 13 s | 4.7 s | **3.6 s** |

Ultimate Taipan, VeriAbs, and VIAP. Fig. 4a directly compares the CPU times spent on tasks of in the subcategory *ReachSafety-Loops*, which is known to contain many tasks that require effort to be spent on generating loop invariants, by both VeriAbs, which was the best verifier in that subcategory, and KI⇜-KIPDR. We observe that for the majority of tasks that were solved by both verifiers, KI⇜-KIPDR is faster than VeriAbs, often by more than an order of magnitude. This shows that the invariant generator KIPDR can be significantly faster than other approaches, depending on the benchmark set. As before, a more in-depth discussion can be found in the technical report [8].

**Comparison against PDR-Based Verification Tools.** The last three columns of Table 1 give an overview over the best configurations of three software verifiers that use adaptations of PDR: For CPAchecker, we selected KI⇜-DF;KIPDR. For SeaHorn, we used the same configuration as submitted by the developers to the 2016 Competition on Software Verification (SV-COMP 2016) [22]. For Vvt, we used the portfolio configuration. We observe that SeaHorn achieves the highest number of correct proofs, but also has a significant amount of incorrect proofs. CPAchecker is the slowest of the three tools and finds fewer proofs than SeaHorn, but CPAchecker has no wrong proofs, and also closely leads in the amount of found bugs. The score-based quantile plot of these results displayed in Fig. 4b visualizes the effects of incorrect results on the computed score. While the graph for SeaHorn is longer, i.e., shows that it solved the most tasks, it is offset to the left by a total penalty of −3 344 points, such that in the end, KI⇜-DF;KIPDR accumulates the highest score because it has a smaller penalty of only −32 points.

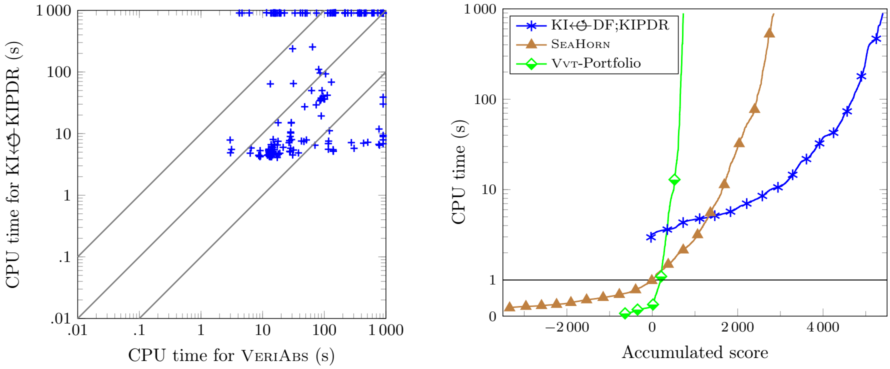

**(a)** Scatter plot comparing the CPU times spent on tasks by VeriAbs and KI←⟲-KIPDR

**(b)** Quantile plot for accumulated score of solved tasks (offset to the left by total penalty from wrong results) showing the CPU time (linear scale below 1 s, logarithmic above) for the successful results of KI←⟲-DF;KIPDR, SeaHorn, and Vvt-Portfolio

Fig. 4: Plots that support the claim that the conclusions of the evaluation are relevant

These results confirm our hypothesis that our previous conclusions are relevant, because they are supported by an implementation that is competitive when compared to the best available PDR-based tool implementations.

### 5 Conclusion

Property-directed reachability (a.k.a. IC3) is a verification approach that is popular and successful in some fields of formal verification (e.g., hardware designs, Horn clauses). Unfortunately, there is a large gap between this success story and the applicability in practical software verification. We are closing this gap by (a) providing a well-engineered implementation of one published adaptation of PDR to software verification, (b) designing and implementing an invariant generator based on the ideas of PDR, and (c) providing an evaluation of all applicable tools and approaches on the largest available benchmark set of C verification tasks. This provides a good foundation as baseline for ongoing research in this area.

The results of our comparative evaluation extend the knowledge about PDR for software verification in the following ways: (1) Our implementation outperforms the existing implementation of PDR (Vvt) and is more precise than the other software verifier that uses PDR (SeaHorn). Thus, our implementation can serve as a reference implementation for further research on PDR for software verification. (2) On most of the programs in the widely used *sv-benchmarks* collection of verification tasks, other techniques are more effective (solve more problems) and more efficient (solve the problems faster). (3) PDR can be an effective and efficient technique for computing invariants that are difficult to obtain: there are programs for which our PDR-based approach is more efficient than the best invariant generator from SV-COMP in the subcategory *ReachSafety-Loops*.

#### 5.1 Data Availability Statement

A replication package for this article including all evaluated implementations and BenchExec is available at Zenodo [9]. Current versions of CPAchecker are available at https://github.com/sosy-lab/cpachecker. The benchmark set of SV-COMP 2018 used in Sect. 4 is available online at https://github.com/sosy-lab/sv-benchmarks/releases/tag/svcomp18 and the dataset from SV-COMP 2019 [4] that we analyzed is available at https://sv-comp.sosy-lab.org/2019/results/results-verified/All-Raw.zip.
